# Remaining useful life prediction of lithium-ion batteries using a novel particle flow filter framework with grey model

**DOI:** 10.1038/s41598-025-86511-z

**Published:** 2025-01-26

**Authors:** Wang Shuai, Li Yiting, Zhou Shoubin, Chen Lifei, Michael Pecht

**Affiliations:** 1 Digital Fujian Internet-of-Things Laboatory of Environmental Monitoring, College of Computer and Cyber Security, Fujian Normal University, Fuzhou, 350117 Fujian China; 2 Huafu Hight Technology Energy Storage Co, Yangzhou, 225699 China; 3 CALCE University of Maryland, College Park, MD 20742 USA

**Keywords:** Lithium-ion batteries, Remaining useful life, Particle flow filter, Particle filter, Energy science and technology, Engineering, Mathematics and computing

## Abstract

Remaining useful life (RUL) prediction is a crucial aspect of the prognostics health management of lithium-ion batteries (LIBs). Owing to the influence of resampling technology, particle degradation is often observed in the particle filter-based RUL prediction of LIBs, resulting in a low prediction accuracy and large uncertainty. In this paper, a novel particle flow filter with the grey model method (GM-PFF) is proposed to forecast the RUL and state of health of batteries. First, the least squares method is employed to obtain the initial values for double exponential empirical model parameters. Subsequently, the grey model is used to predict the current cycle capacity of LIBs as an observation value for the particle flow filter, solving the inaccurate estimation problem of the state of particle flow filter observation values, and the particle flow filter method is employed to update model parameters. Finally, a test dataset is divided into early, middle, and late stages to predict the RUL of LIBs and obtain the probability distributions. On the CALCE and NASA PCoE LIB dataset, GM-PFF reduces RMSE by 1% compared to PFF, exhibiting a higher prediction accuracy and effectively addressing the particle degradation problem.

## Introduction

Owing to their large capacity and long lifespan, lithium-ion batteries (LIBs) are commonly used in wearable devices, household appliances, medical equipment, aerospace applications, energy storage, and other fields^1,2^. With the continuing expansion of global industries, LIBs, as efficient and reliable energy storage devices, are increasingly used in various industries, such as new energy vehicles, large energy storage arrays, and transmission systems^3^. Concurrently, with the increasing demand for clean energy, LIBs have also received increasing attention in areas such as energy storage and smart grids. Undoubtedly, with the continuous development of technology, the performance of LIBs will be further improved, and their application fields will continue to expand.

With the increasing use of LIBs, their capacity decline and loss have become inevitable concerns. Solid electrolyte interface (SEI) film growth, electrode material dissolution, and metallic Li deposition primarily occur during LIBs cycles^4,5^. Under normal circumstances, the chemical properties of the internal electrodes and electrolytic materials of LIBs deteriorate with the increase in usage, resulting in increased internal side reactions and diminished battery capacity. The decline of LIB capacity can be used as an indicator to evaluate the remaining useful life (RUL) of an LIB^6^. The RUL of LIBs refers to the number of charging and discharging cycles that batteries experience when their available capacity decays to the end of life (EoL) capacity. Generally, when the available capacity of a battery decays to 70% or 80% of its rated capacity, it is considered to have reached its EoL^7^. If the health status of an LIB is not monitored, it can adversely affect the production and life. For example, the aging of LIBs has led to catastrophic accidents, such as fires and explosions in electric vehicles. Therefore, understanding and monitoring the state of health of LIBs and accurately predicting the RUL of LIBs under different conditions ensures the safe and reliable operation of the system while maximizing the use of the remaining value of the battery^8^.

To predict the RUL of lithium-ion batteries, scholars strive to identify the patterns of capacity degradation. However, the capacity fading of LIBs is nonlinear, making it challenging to establish an accurate mathematical model. Two approaches exist to predict the RUL of LIBs: model-driven and data-driven approaches^9^. The model-driven method establishes a mathematical model to predict the RUL of LIBs; however, it requires a thorough analysis of the internal degradation mechanisms of LIBs. The methods of optimizing model parameters, such as using the Kalman filter (KF) or particle filter (PF), can improve the prediction accuracy. Nevertheless, owing to the complexity and various influences of the degradation phenomena of LIBs, the accuracy of predictions using these methods may be limited. The data-driven method starts by collecting the historical capacity decline data of LIBs. Subsequently, the method uses machine learning techniques, such as support vector regression (SVR)^10^, neural networks (NNs)^11^, and Gaussian process regression (GPR)^12^, to mine hidden data and establishes the mathematical relationship between known and unknown data, thereby predicting the RUL of LIBs. Owing to the extensive data required for training machine learning methods, acquiring a significant amount of data in real-world applications to train precise models is challenging.

As early as in 2008, Saha and Goebel^13^ proposed the Bayeux Adams Theorem, a battery utilization computer application, to diagnose and predict the uncertainty of battery health and establish the RUL of LIBs, which pioneered the prediction and development of particle filter in the RUL of LIBs. LIBs have a higher energy density and longer lifespan than conventional batteries, making them the power source of choice for many modern electronic devices. The PF is an approximate Bayesian filter algorithm based on Monte Carlo sampling. It is suitable for nonlinear and non-Gaussian systems. The main idea of the PF is to extract random state particles from the posterior distribution to represent the distribution. The likelihood of extraction increases with the particle weight. However, after many iterations, only a few samples among a large number of particle samples have a higher weight, while low-weight samples still occupy computing resources, which is known as the particle depletion phenomenon^14^. Qiang Miao et al. introduced the unscented PF (UPF) into the prediction of battery RUL. The algorithm combines the PF and unscented KF (UKF) to improve the prediction accuracy of the PF algorithm^15^. Qiu et al. proposed using the improved cuckoo search algorithm to iteratively optimize particles and force them to move toward the maximum likelihood region, thus relieving the particle degeneracy phenomenon during filtering and improving the prediction accuracy^16^. Shuai Wang et al. proposed a prediction method based on the interacting multi-model PF, which can be used to determine the uncertain distribution of RUL^17^.

Daum et al. proposed a particle flow filter (PFF) called the Daum–Huang filter (DHF) to solve particle degradation; the filter gives each particle the same weight, without resampling, and does not rely on suggested density functions. Introducing the concept of homotopy theory, the particles flow from the prior probability distribution to the posterior probability distribution^18^. Generally, the PFF is approximately two orders of magnitude more accurate than the extended KF (EKF) or UKF in dealing with nonlinear problems. To achieve the same accuracy as that of the standard PFF, the PFF requires a reduction in number of particles by three orders of magnitude^19^. The flow strategy plays a key role in the PFF method. Different flow strategies result in different forms of particle flow. The information of particles can be fully utilized by designing a suitable flow strategy, improving the accuracy of prediction. In previous studies, various flow strategies, such as the IPF filter, NZDDH filter, and exacted DHF (EDHF), have been proposed^20–26^. Choi et al. proved that the performances of the IPF and EDH filters are better than those of well-known algorithms (bootstrap PF, auxiliary PF, EKF, and UKF) under linear, nonlinear, Gaussian, non-Gaussian, and different state space dimensions and particle numbers^27^. Mori et al. proposed an adaptive step-size PFF to deal with the rigid flow caused by solving differential equations, enabling a smoother particle flow from the prior distribution to the posterior distribution^28^. Different particle flow strategies can be selected and adjusted based on the characteristics of specific problems to obtain the best prediction effect.

Considering the excellent performance of the PFF in nonlinear dynamic systems, this study aims to apply the EDHF to estimate the parameters of the double-exponential model of lithium-ion battery capacity decay. The main contributions of this study are as follows:

1. The PFF is used for predicting the RUL of LIBs. Experiments are carried out on CALCE data sets, evaluation indicators use the root mean square error, absolute error and precision index. The results show that the method can greatly improve the accuracy and probability density distributions of LIB RUL prediction.
2. Combining the grey model with the PFF leverages the advantage of the grey model in providing relatively reliable predictions even in scenarios with limited sample data. Particle flow filtering, similar to traditional particle filtering, heavily relies on the observation equation for prediction accuracy. The predictive outcomes of the grey model are introduced into the PFF as observation states, and the particle weights are updated. The integration enables to better capture dynamic changes in the system, enhancing the accuracy of future state predictions.

The remainder of this manuscript is organized as follows. Section “Methodology” introduces the principles of the grey model and PFF. Section 3 introduces the experiment. The experimental results are presented and discussed in Sect. 4. Finally, Sect. 5 provides the summary of the study and the future scope of work.

## Methodology

### Grey model

The grey model was first proposed by Deng in 1982^29^. It lies between the white model and black model and represents a system with partially known internal information and partially unknown information^30^. The grey model has unique advantages in predicting the data quality under extremely small sample sizes, with relatively good prediction effectiveness. The most typical one is GM (1,1); the process of establishing GM (1,1) is as follows^31^.

For a set of historical data,

1 $${Z^{(0)}}=[{z^{(0)}}(1),\;{z^{(0)}}(2), \ldots ,\;{z^{(0)}}(n)].$$

The first-order accumulated generation order (1-AGO) of the historical array sequence is expressed as

2 $${Z^{(1)}}=\left[ {{z^{(0)}}(1),\;\sum\limits_{{i=1}}^{2} {{z^{(0)}}(i)} , \ldots ,\sum\limits_{{i=1}}^{n} {{z^{(0)}}(i)} } \right].$$

The whitening differential equation is given by

3 $$\frac{{d{Z^{(1)}}}}{{dt}}+a{Z^{(1)}}=b,$$

where $\alpha$ denotes the development grayscale, and $\beta$ denotes the internal generation control grayscale; they conform to

4 $$\begin{gathered} [\alpha ,\beta ]^{T} = (B^{T} B)^{{ - 1}} B^{T} Y, \hfill \\ B = \left[ {\begin{array}{*{20}c} { - 0.5 \times (z^{{(1)}} (2) + z^{{(1)}} (1))} & 1 \\ { - 0.5 \times (z^{{(1)}} (3) + z^{{(1)}} (2))} & 1 \\ \vdots & \vdots \\ { - 0.5 \times (z^{{(1)}} (n) + z^{{(1)}} (n - 1))} & 1 \\ \end{array} } \right] \hfill \\ Y = [\begin{array}{*{20}c} {z^{{(1)}} (2)} & {z^{{(1)}} (3)} & {\begin{array}{*{20}c} \cdots & {z^{{(1)}} (n)} \\ \end{array} } \\ \end{array} ]^{T} \hfill \\ \end{gathered}$$

The restored data and predicted values are as follows:

5 $$\begin{gathered} {{\hat {z}}^{(1)}}(t+1)=[{z^{(0)}}(1) - b/a]{e^{ - at}}+b/a, \hfill \\ {{\hat {z}}^{(0)}}(t+1)={{\hat {z}}^{(1)}}(t+1) - {{\hat {z}}^{(1)}}(t)=(1 - {e^a})[{z^{(0)}}(1) - b/a]{e^{ - at}}. \hfill \\ \end{gathered}$$

### Particle flow filter

In the dynamic nonlinear system model, let ${{\text{X}}_k}$represent the state vector of the target at time k and${{\text{Z}}_k}$denote the measurement value. Accordingly, the state equation and measurement equation are

6 $$\begin{gathered} {X_k}={g_k}\left( {{X_{k - 1}},{\mu _k}} \right) \hfill \\ {Z_k}={h_k}\left( {{X_k},{\nu _k}} \right), \hfill \\ \end{gathered}$$

where ${g_k}$ represents the state transition function, and ${\mu _k}$ denotes the process noise. Moreover, ${{\text{h}}_k}$ represents the observation function, and ${\nu _k}$ denotes the measurement noise.

Solving the Bayesian filter usually entails two steps:

(1) Prediction: Bayes’ theorem is used to infer the next state of the system based on the dynamic model and prior information of the system. The prediction step relies on the current state estimation and the system model, advancing and forecasting the state using the state transition equation:

7 $$p({X_k}\left| {{Z_{1:k - 1}})} \right.=\int {p({X_{k - 1}}\left| {{Z_{1:k - 1}})} \right.p({X_k}\left| {{X_{k - 1}})} \right.} d{X_{k - 1}}.$$

(2) Update: Considering the observed data, an estimate of the state of the system is updated by comparing the observed data with the predicted estimate. The update step is based on Bayes’ theorem, using the observed data to correct the uncertainty of the forecast estimates.

8 $$p({X_k}\left| {{Z_{1:k}})} \right.=\frac{{p({X_k}\left| {{Z_{1:k - 1}})} \right.p({Z_k}\left| {{X_k})} \right.}}{{\int {p({X_k}\left| {{Z_{1:k - 1}})} \right.p({Z_k}\left| {{X_k})} \right.} d{X_k}}},$$

where $p({X_k}\left| {{X_{k - 1}})} \right.$ represents the state transition density function, and$p({Z_k}\left| {{X_k})} \right.$ represents the likelihood function. The estimate of the system state can be continuously and iteratively updated by alternating the prediction and updating steps, enabling efficient inference and prediction of the system state^32^. While estimating the system state, the Bayesian filter method also provides quantification of the estimation uncertainty, making the inference of the system state more reliable and accurate.

The PFF introduces the homotopy principle in the topology to move particles from prior distribution to posterior distribution^33^. According to the Bayesian rule, the posterior probability density function (PDF) is calculated as

9 $$p({X_k}\left| {{Z_{1:k}})} \right.=\frac{{p({X_k}\left| {{Z_{1:k - 1}})} \right.p({Z_k}\left| {{X_k})} \right.}}{{p({Z_k}\left| {{Z_{k - 1}})} \right.}},$$

where $p({Z_k}\left| {{Z_{k - 1}})} \right.=\int {p({X_k}\left| {{Z_{1:k - 1}})} \right.p({Z_k}\left| {{X_k})} \right.} d{X_k}$ is the normalization coefficient. The PF is a nonlinear filter algorithm based on the Monte Carlo method, which approximates the posterior probability distribution of a system through a series of randomly generated particles. The larger the particle weight, the closer it is to the real state. During the resampling process, particles with significant weights are more likely to be resampled, resulting in a loss of particle diversity.

The PFF assigns the same weight $\omega _{k}^{j}$ to ${N_p}$ particles:

10 $$\omega _{k}^{j}=\frac{1}{{{N_p}}}.$$

For convenience, let $m\left( {{X_k}} \right)$ and $n\left( {{X_k}} \right)$represent the likelihood function ${\text{p(}}{{\text{Z}}_k}\left| {{X_k})} \right.$ and the prior probability density, respectively. The logarithmic homotopy function $\log p({X_k},\lambda )$ can be defined as

11 $$\log p({X_k},\lambda )=\log m({X_k})+\lambda \log n({X_k}) - \log K\left( \lambda \right),$$

where $\lambda$ signifies a pseudo-discrete time, ranging from 0 to 1. The normalization coefficient is $K\left( \lambda \right)=\int {g\left( {{X_k}} \right)} h{\left( {{X_k}} \right)^\lambda }dX$. When $\lambda$ = 0, the particle flow is in the prior distribution state; while $\lambda$ changes from 0 to 1, the particles move gradually, until $\lambda$ = 1, at which point the particles move to the appropriate posterior distribution area. It is assumed that the particle flow obeys the Ito stochastic differential equation^34^:

12 $${\text{dX=}}f\left( {X,\lambda } \right)d\lambda +\sigma \left( {X,\lambda } \right)d{W_\lambda },$$

where$f\left( {X,\lambda } \right)$ represents the flow vector, and $\sigma \left( {X,\lambda } \right)$ is the diffusion coefficient. Based on different assumptions, different solutions to Eq. (12) exist, and the precise PFF is a well-known solution method^35,36^. The EDH assumes that both the prior distribution and the likelihood distribution obey Gaussian distribution, and the precise PF can be expressed as

13 $$f\left( {X,\lambda } \right)=A\left( \lambda \right)X+b\left( \lambda \right),$$

where

14 $$\begin{gathered} A\left( \lambda \right)= - \frac{1}{2}P{H^T}{\left( {\lambda HP{H^T}+R} \right)^{ - 1}}H \hfill \\ b\left( \lambda \right)=\left( {I+2\lambda A\left( \lambda \right)} \right)\left[ {A\left( \lambda \right)\overline {X} +\left( {I+\lambda A\left( \lambda \right)} \right)P{H^T}{R^{ - 1}}z} \right]. \hfill \\ \end{gathered}$$

Here, *R* denotes the measurement noise variance matrix, *P* represents the prior covariance matrix, and H can be obtained through the first-order linearization of the measurement function. The state update of the j_th_ particle is

15 $$X_{k}^{j}=X_{{k - 1}}^{j}+\Delta \lambda (AX_{{k - 1}}^{j}+b).$$

## Experimental design

### Lithium-ion battery dataset

**Fig. 1:**
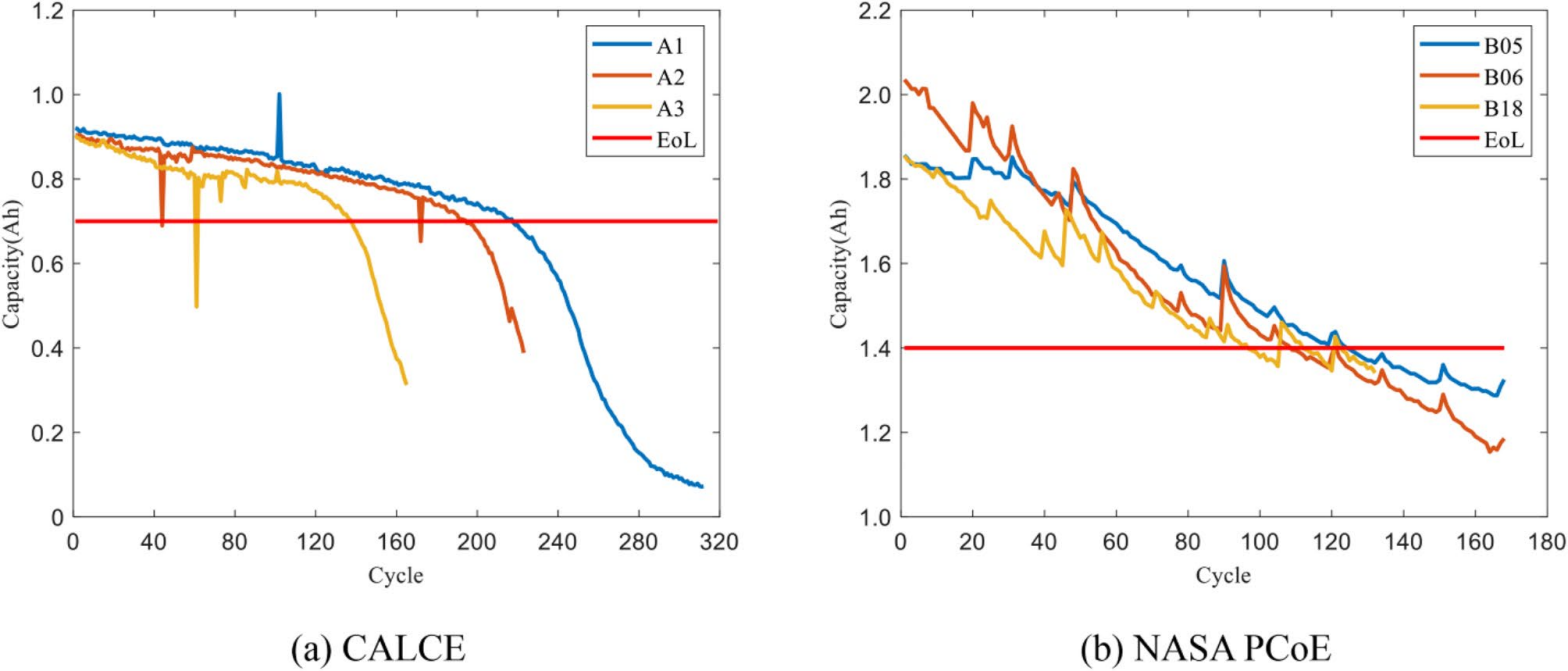
Capacity degradation curve of lithium-ion batteries (LIBs).

The lithium-ion batteries data used in this paper come from the Center for Advanced Life Cycle Engineering (CALCE) at the University of Maryland, College Park and National Aeronautics and Space Administration Prognostics Center of Excellence (NASA PCoE). LIBs operated under three conditions of charging, discharging, and EIS impedance at room temperature (24 °C). The termination life threshold of the battery is set at 70% of the rated capacity, which is 1.4Ah. The CALCE LIBs started constant current discharge from 4.2 V until the voltage droped to 2.5 V. They had a similar number of cycles to the previous three groups of batteries. The EoL of the batteries is 0.7Ah. The capacity degradation trends of two LIBs datasets are depicted in Fig. 1.

The end of life of batteries B05, B06, and B18 are 125, 109, and 112 cycles, respectively, while the EoL of batteries A1, A2, and A3 are 218, 197, and 144 cycles, respectively. Among these batteries, B06 has the shortest end of life, only 109 cycles. In order to further explore the laws of battery performance degradation and achieve early prediction of battery remaining useful life, this experiment set different life stages for more detailed observation and analysis, with 70 cycles set as the early stage, 80 cycles set as the mid stage, and 90 cycles set as the late stage.

### Particle flow filter framework with grey model

The battery capacity calculation formula is as follows:

16 $$Q=\int {I\left( t \right)} dt.$$

During the cycle use of LIBs, the capacity gradually decreases. The overall trend is initially fast and then slow. Once the capacity drops to a certain critical value, the battery can no longer be used. Currently, the recognized threshold is 70% of the rated capacity of the battery. According to the existing physical knowledge, the mapping relationship between the capacity of LIBs and the charge–discharge cycle is established, that is, the capacity decline model of LIBs:

17 $${\text{Q}}=a * \exp (b * k)+c * \exp (d * k),$$

where *a*,* b*,* c*, and *d* are model parameters, and *k* is the number of charge and discharge cycles of the lithium battery. This model is also called a double-exponential empirical decay model.

The state transition equation and measurement equation of the LIB system are as follows:

$${{\text{X}}_k}=\left[ {{a_k},{b_k},{c_k},{d_k}} \right],$$

18 $$\left\{ \begin{gathered} {a_k}={a_{k - 1}}+{\omega _a},{\omega _a}\sim N\left( {0,{\sigma _a}} \right) \hfill \\ {b_k}={b_{k - 1}}+{\omega _b},{\omega _b}\sim N\left( {0,{\sigma _b}} \right) \hfill \\ {c_k}={c_{k - 1}}+{\omega _c},{\omega _c}\sim N\left( {0,{\sigma _c}} \right) \hfill \\ {d_k}={d_{k - 1}}+{\omega _d},{\omega _d}\sim N\left( {0,{\sigma _d}} \right) \hfill \\ \end{gathered} \right.$$

19 $${{\text{Q}}_k}={a_k} * \exp ({b_k} * k)+{c_k} * \exp ({d_k} * k)+{v_k},$$

In order to obtain accurate parameter estimation for the battery experience degradation model, the Matlab curve fitting toolbox was first used to fit six sets of experimental data from two datasets, obtaining the parameters a, b, c, and d of empirical formula (19) (see Table 1). Then take the mean parameter value of each dataset as the initial parameter for the least squares method.

**Table 1:** Model parameter settings.

	a	b	c	d	Mean
B05	1.979	-0.00272	-0.1697	-0.06942	0.43429
B06	1.57	-0.00558	0.489	0.000945	0.513592
B18	1.858	-0.00292	0.000191	0.0482	0.475869
A1	-0.1004	0.008761	0.98	0.001297	0.222415
A2	-9.86E-07	0.05752	0.8983	-0.00083	0.238746
A3	-1.53E-05	0.06296	0.8757	-0.00094	0.234426

The probability density function (PDF) describes the probability distribution of a battery reaching the failure threshold. The PDF of predicted RUL can be further calculated by

20 $$PDF=\sum\limits_{{j=1}}^{{{N_p}}} {{\omega ^j}\delta (RUL - RU{L^j})} .$$

**Fig. 2:**
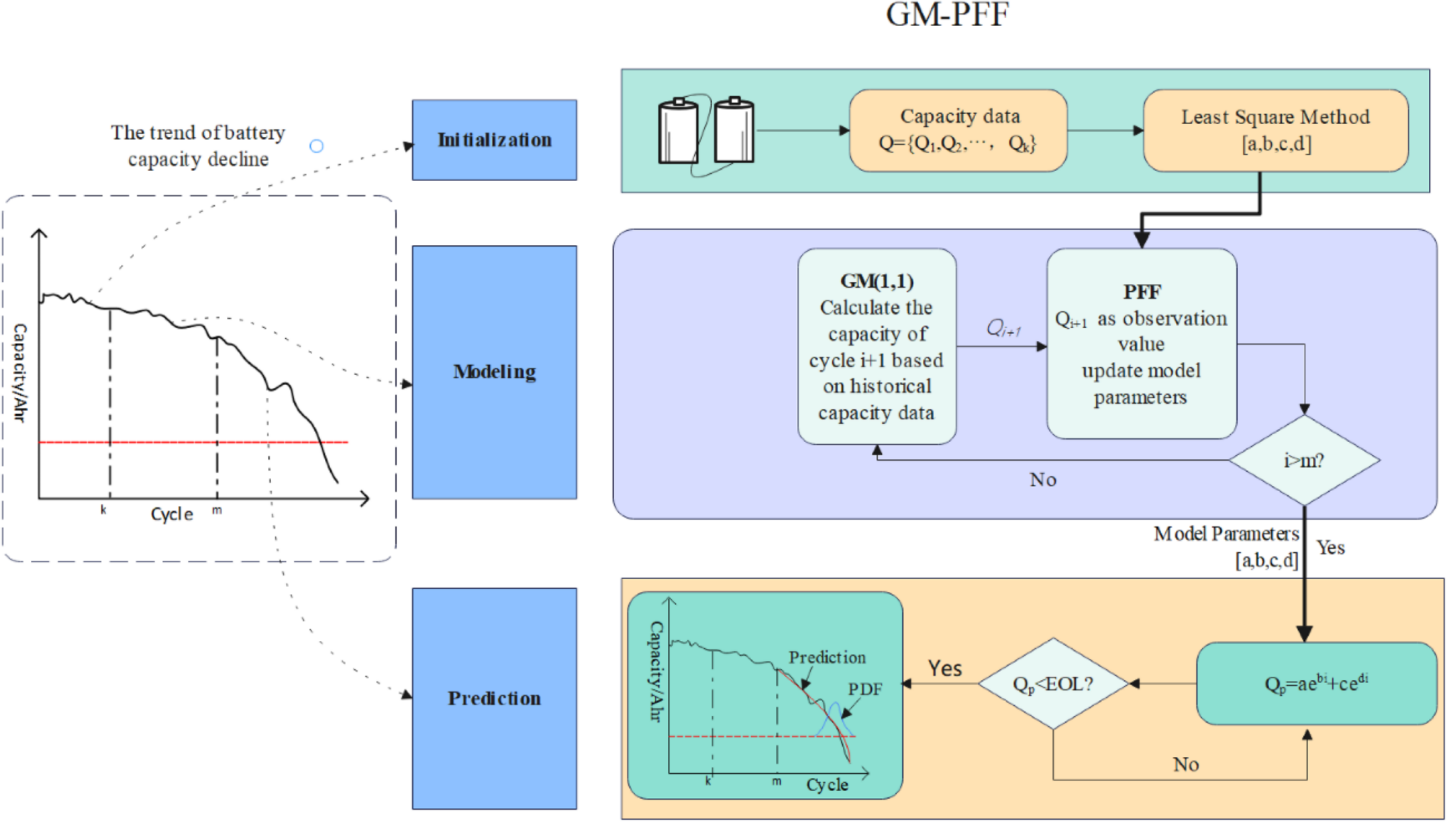
GM-PFF framework for RUL prediction.

The framework of the proposed GM-PFF is depicted in Fig. 2.

The specific implementation processes for battery RUL prediction are as follows:

Step 1: Initialize the number of particles ${N_p}$ is 100, measurement noise ${\omega _a}\sim N\left( {0,1e - 6} \right)$, ${\omega _b}\sim N\left( {0,1e - 6} \right)$, ${\omega _c}\sim N\left( {0,1e - 6} \right)$, ${\omega _d}\sim N\left( {0,1e - 6} \right)$ and observation noise ${\nu _k}\sim N\left( {0,1e - 3} \right)$; fit the parameters of the degradation model for the first *k* cycles of the battery capacity using the least squares method; set the starting time ST = k+1; obtain the actual capacity ${{\text{Q}}_i}\;(i=k+1,\;k+2, \ldots ,\;m)$ as training data; and set the gray model sliding window t.

Step 2: Obtain the particle distribution, use the grey model to predict the battery capacity of the k + 1 cycle as an observation value for the PFF, find the Jacobian matrix H, and obtain A and b introduced in Sect. 1. Particles flow to a posterior distribution, update particle distribution.

Step 3: Set the prediction phase after the m + 1th cycle; if cycle i < m, repeat Step 1.2 and set the capacity failure threshold as FT.

Step 4: Use degradation models to predict battery capacity Q. In comparing Q and FT, if Q > FT, let m = m + 1 and repeat the prediction until Q < FT.

Step 5: Obtain the RUL of LIBs using RUL = m-ST-k and calculate the remaining lifespan predicted for each particle as a PDF.

### Evaluation index

Root mean square error (RMSE): The error between actual battery capacity and predicted battery capacity

13 $${\text{RMSE}}=\sqrt {\frac{1}{N}\sum\limits_{{i=1}}^{N} {{{\left( {{Q_{real}} - {Q_{prediction}}} \right)}^2}} } .$$

Absolute error (AE): The absolute value of the difference between the actual remaining life of the battery and predicted remaining life.

14 $${\text{AE=}}\left| {{\text{RU}}{{\text{L}}_{true}} - {\text{RU}}{{\text{L}}_{prediction}}} \right|$$

Precision index (PI): The relative width of the probability density interval. And $\sup (RUL)$ and $\inf (RUL)$ represent the upper and lower bounds of the probability density interval, respectively.

15 $${\text{PI=}}\sup (RUL) - \inf (RUL)$$

## Results and discussion

Owing to the nonlinear and stochastic characteristics of LIB capacity degradation trajectories, the grey model demonstrates superiority in handling small-sample nonlinear systems. It can be utilized to provide rough estimates of the LIB capacity.

**Fig. 3:**
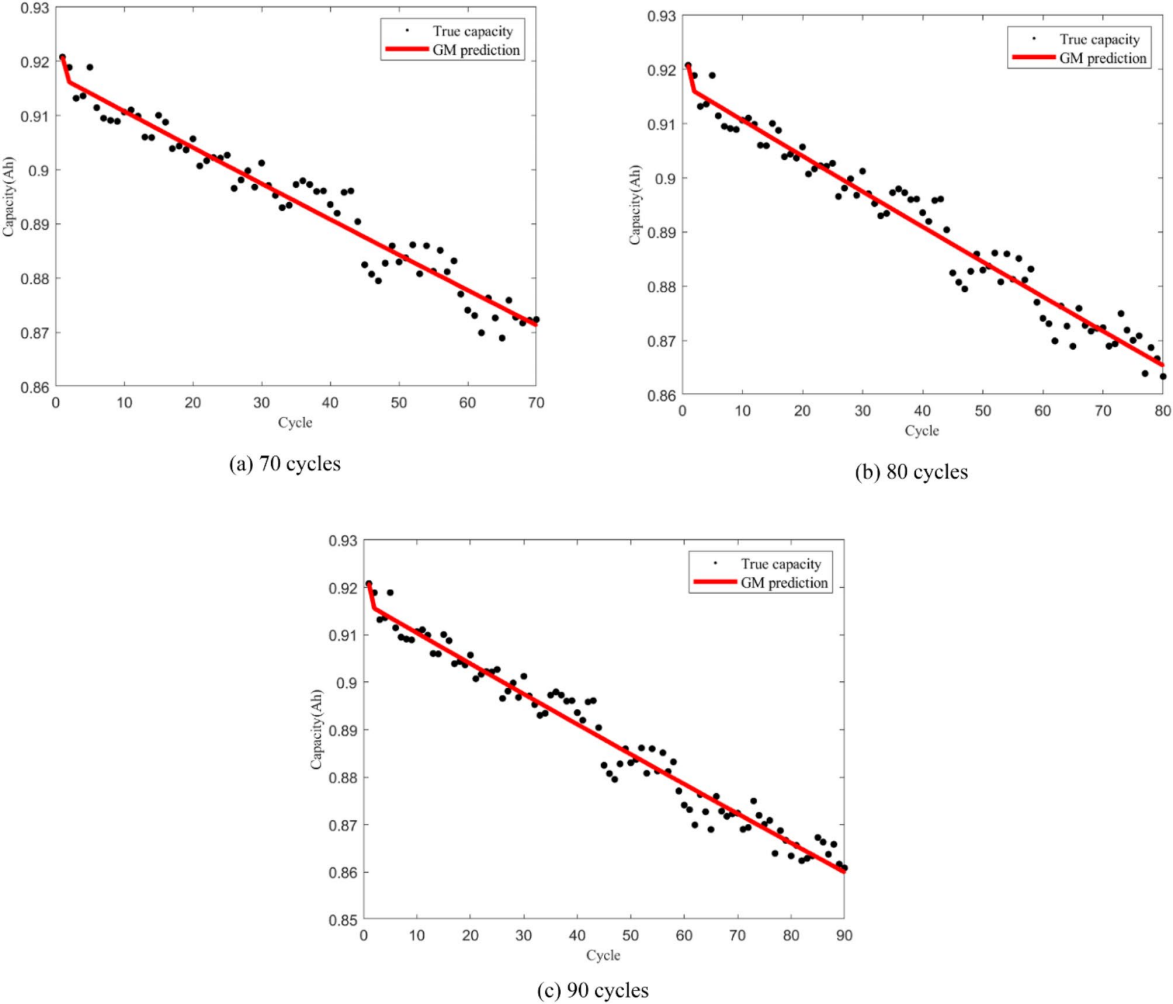
Fitting Results of GM (1,1) for battery A1.

Considering battery A1 as an example, the training data include those of 70, 80, and 90 cycles, and the capacity prediction results of the grey model are depicted in Fig. 3. Evidently, the GM (1,1) prediction trajectory approximates a straight line, making it suitable for predicting the degradation trend of the LIB capacity. However, its performance in predicting random terms is relatively weak.

The prediction results of GM (1,1) for three battery groups are presented in Table 2. $\alpha$ and $\beta$ are parameter estimates of the grey model. To validate the accuracy of grey predictions, we introduce the posterior difference ratio c; a smaller c indicates a higher prediction accuracy. Generally, c values below 0.35, 0.5, and 0.65 signify high model accuracy, qualified accuracy, and basically qualified accuracy, respectively. Conversely, a c value that exceeds 0.65 indicates inadequate model accuracy. For battery A1, all c values are below 0.5, indicating a relatively high model accuracy and closeness of GM (1,1) predictions to the actual values. However, for battery A2, we observe that the later-stage c value is below 0.65, indicating basically qualified accuracy, while the early-stage c value exceeds 0.65, suggesting insufficient accuracy of GM (1,1) during that period. Overall, the c values for battery A2 fall between 0.5 and 0.65, indicating a moderate level of model accuracy.

This implies that GM (1,1) is feasible for predicting LIB capacity, particularly for battery A1, for which the model performs robustly. However, it is crucial to note that GM (1,1) is sensitive to historical data, particularly when considering degradation trends, where the influence of random terms is significant, potentially resulting in relatively poor model prediction effectiveness.

**Table 2:** Performance evaluation of GM (1,1) model.

Battery cycle		$\alpha$	$\beta$	c
A1	70	0.001	0.917	0.05
	80	0.001	0.917	0.039
	90	0.001	0.917	0.032
A2	70	0	6.897	0.663
	80	0	7.894	0.645
	90	0	8.892	0.603
A3	70	0	11.905	0.505
	80	0	13.897	0.527
	90	0	14.89	0.545

**Fig. 4:**
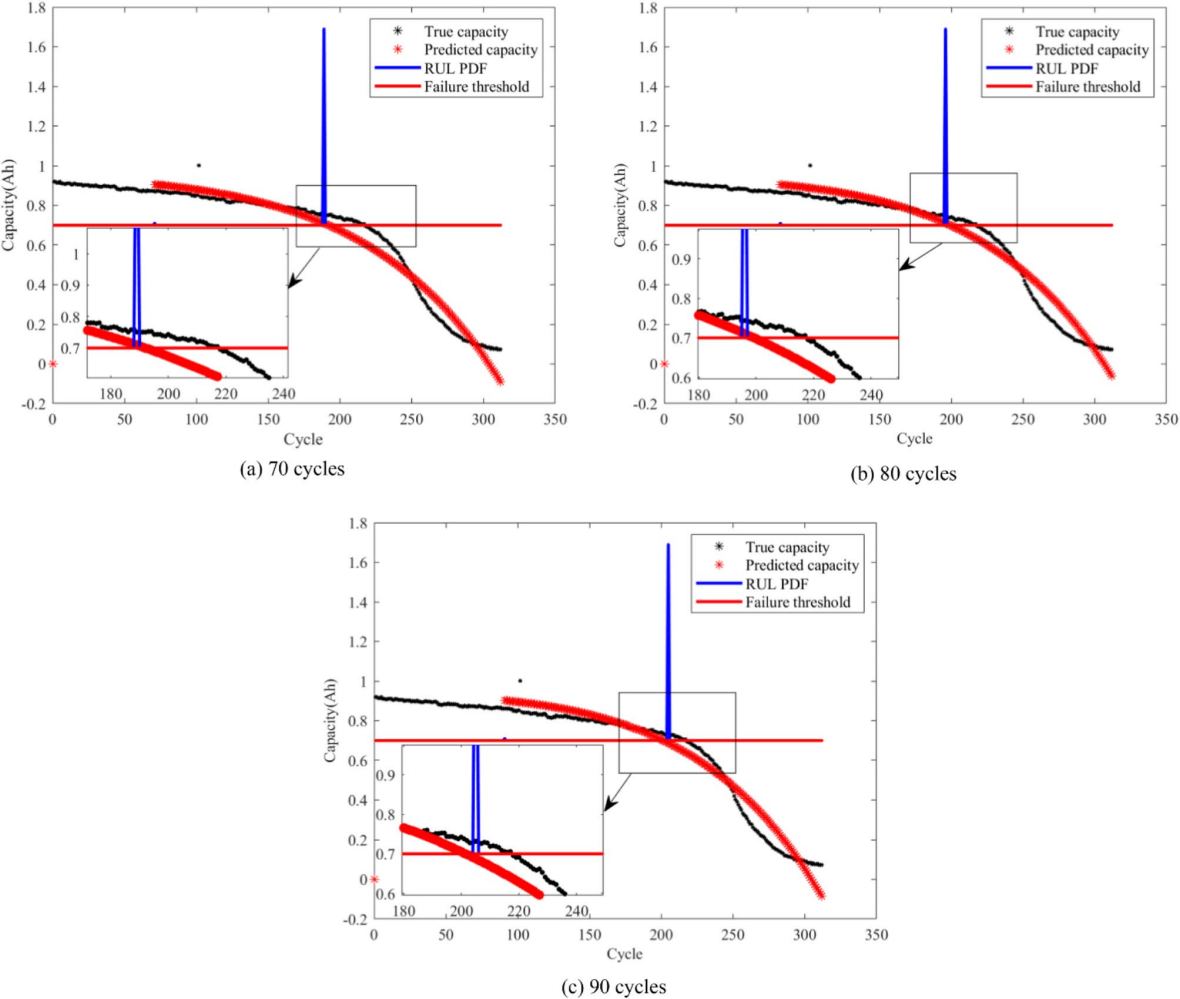
Prediction results for capacity degradation trend of battery A1.

**Fig. 5:**
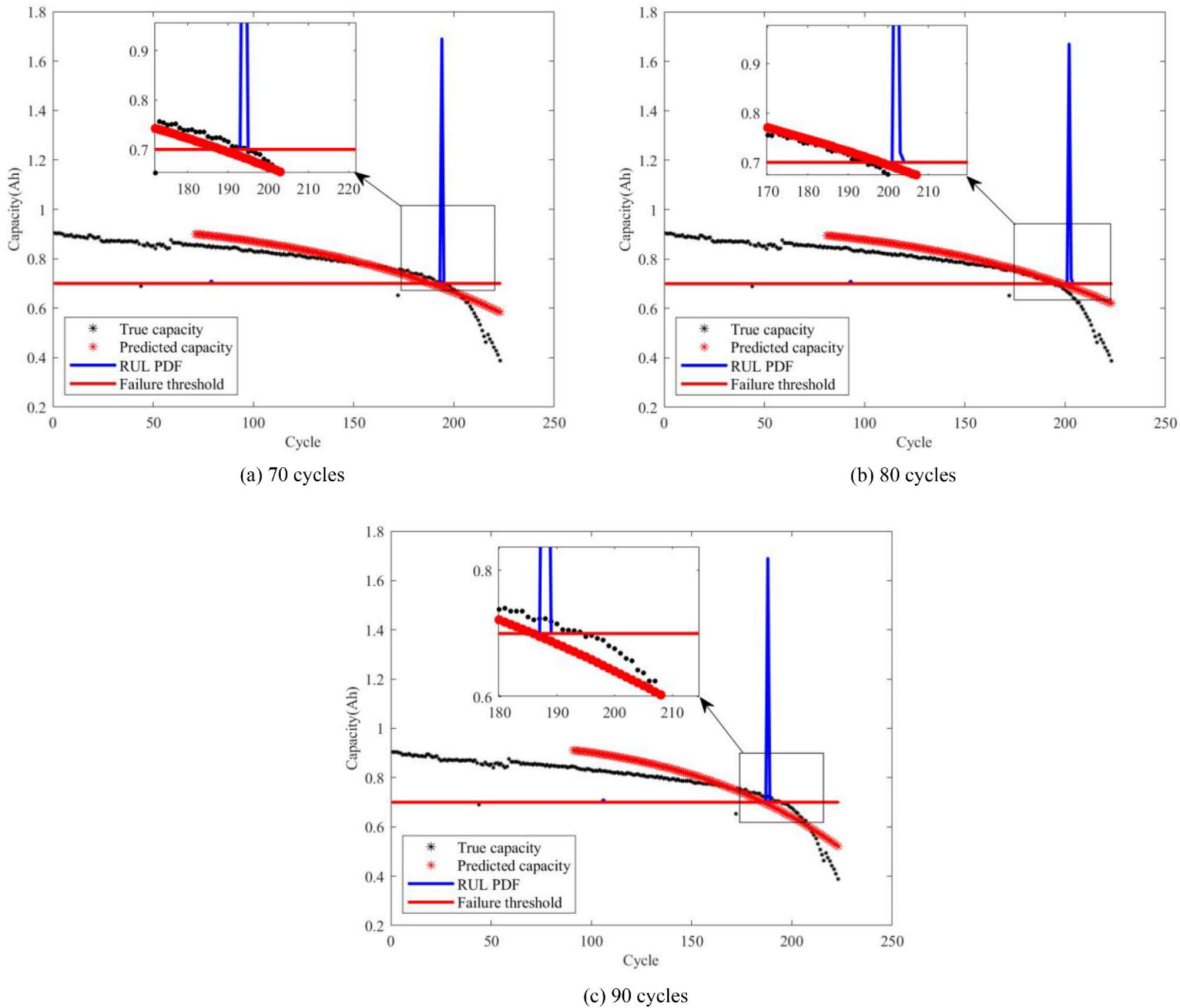
Prediction results for capacity degradation trend of battery A2.

**Fig. 6:**
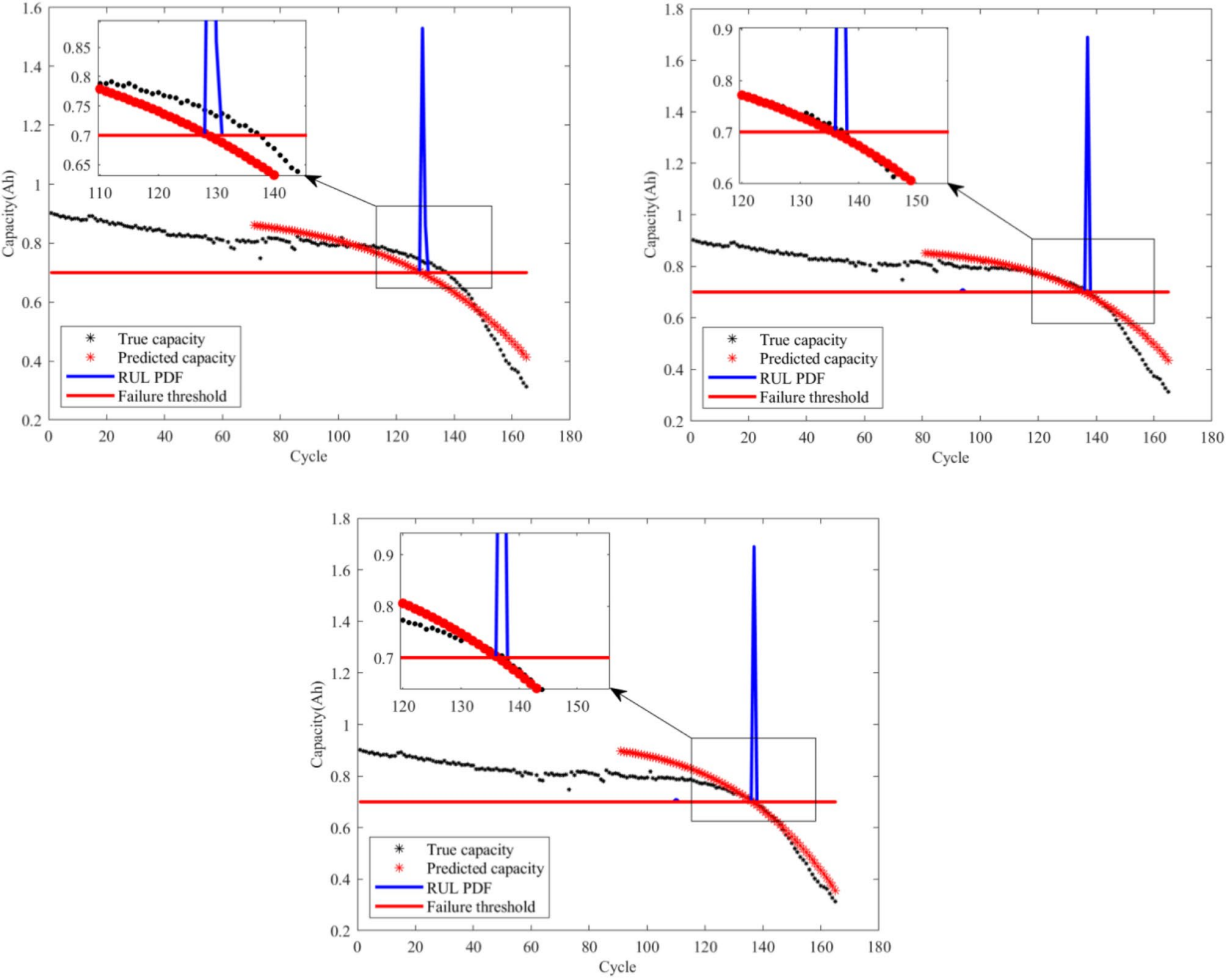
Prediction results for capacity degradation trend of battery A3.

**Fig. 7:**
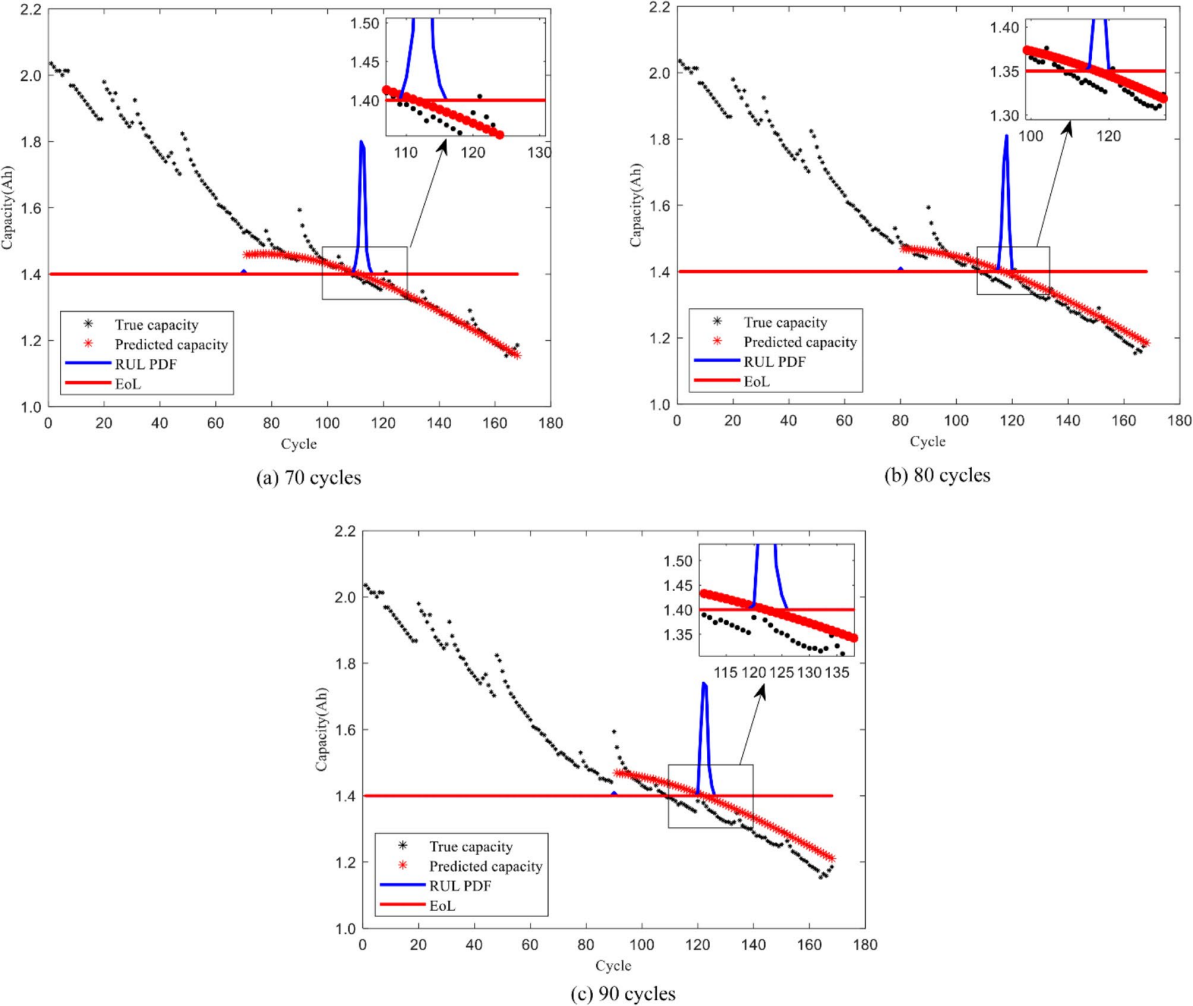
Prediction results for capacity degradation trend of battery B06.

From Figs. 4, 5, 6 and 7, it is evident that the GM-PFF predictions closely align with the actual capacity degradation trends. The predicted endpoints of battery life closely match the actual endpoints, and the PI primarily concentrates around the predicted endpoints. The width of CALCE spans 2–3 cycles, while NASA PCoE’s width is 5–7 cycles. This suggests a concentrated particle distribution, indicating the accuracy of the proposed model in predicting the lifespan of LIBs.

Furthermore, we established three key prediction stages in the graph, corresponding to different developmental phases of battery life. Initially, 70 cycles serve as the early prediction stage, aiming to capture the initial performance degradation trends of the battery. Subsequently, 80 cycles represent the mid-term prediction stage with the aim of delving deeper into understanding the mid-term patterns of battery performance degradation. Finally, 90 cycles signify the late-stage prediction, aiming to accurately capture the characteristics of batteries that approach failure.

The black markers in the graph depict the actual evolution of LIB capacity, while the red markers represent the corresponding predictions of the remaining capacity using the GM-PFF model. This clear comparison demonstrates the accurate prediction of the battery capacity degradation trend by GM-PFF. The red solid line indicates the battery failure threshold at the 0.7 Ah capacity level of CALCE and 1.4 Ah capacity level of NASA PCoE. The blue solid line represents the PI that predicts the distribution of possible battery lifespans.

The graph analysis clarifies that the predictions of the GM-PFF model for the capacity degradation trend of LIBs closely match the actual situation. The predicted endpoints of battery life align closely with the actual endpoints, and the concentration of the PI around the predicted endpoints, with a width of 2–3 or 5–7 cycles, indicates a focused particle distribution. This means that the proposed model provides a relatively accurate estimate of the RUL of LIB, and reduces the range of uncertainty, providing greater support for decision-making.

A comparison of predictions between the GM-PFF and PFF on two datasets is presented in Tables 3 and 4. Both methods maintain consistency in the number of particles (100) as well as the experimental environment and parameter settings. From the perspective of RMSE, GM-PFF exhibits lower error values in all battery predictions. Taking the early stage of battery A1 as an example, the RMSE value of GM-PFF reached 0.0569, while the RMSE value of PFF was 0.0827, reducing the error of GM-PFF by about 31%. GM-PFF reduces RMSE error by more than 3% on the CALCE dataset and by more than 6% on the NASA PCoE dataset. This data clearly indicates that GM-PFF has a significant advantage in prediction accuracy. In terms of AE, taking the later stage of battery B06 as an example, the AE value of GM-PFF is only 3, far lower than PFF’s 14, and the error has been reduced by about 79%. GM-PFF has reduced AE prediction errors in both datasets, demonstrating its higher accuracy and stability in predicting the remaining battery life. The PI value of GM-PFF is also relatively small, which means that the uncertainty of its prediction results is lower. We noticed that the errors in predicting B06 and B18 in GM-PFF at the beginning of the 90th cycle were relatively large. This is due to the capacity recovery phenomenon of the battery at the 90th cycle, which caused a deviation between the observed values and the predicted values, affecting the estimated model parameters and increasing the error. Due to the trend of the exponential empirical model, it may cause errors in predicting trends on some batteries. As the predicted data increases, the prediction error of GM-PFF decreases on CALCE batteries. GM-PFF is more reliable in predicting battery capacity degradation and can provide more accurate guidance for battery management and maintenance.

In summary, the GM-PFF demonstrates a remarkable predictive performance in handling the nonlinear and complex nature of LIB capacity degradation. The results of this study indicate that the GM-PFF is highly applicable for addressing battery capacity degradation problems characterized by randomness and complexity.

**Table 3:** Comparison results of GM-PFF and PFF in the CALCE battery dataset.

Batteries cycle		A1			A2			A3		
		70	80	90	70	80	90	70	80	90
GM-PFF	RMSE	0.0569	0.0572	0.0581	0.0477	0.0613	0.0499	0.0443	0.0503	0.0512
	AE	25	18	15	6	4	8	9	2	1
	PI	2	2	2	2	3	2	3	2	2
PFF	RMSE	0.0827	0.0702	0.0756	0.0571	0.0655	0.0598	0.0510	0.0519	0.0533
	AE	15	6	7	7	11	7	3	7	3
	PI	3	2	2	3	3	2	3	2	2

**Table 4:** Comparison results of GM-PFF and PFF in the NASA PCoE battery dataset.

Batteries cycle		B05			B06			B18		
		70	80	90	70	80	90	70	80	90
GM-PFF	RMSE	0.0382	0.0339	0.0396	0.0288	0.0246	0.0426	0.0492	0.0474	0.0495
	AE	6	3	2	3	5	3	1	3	8
	PI	3	3	6	6	6	6	2	2	2
PFF	RMSE	0.0418	0.0492	0.0495	0.0461	0.0396	0.0454	0.0529	0.0526	0.0571
	AE	6	13	6	3	8	14	11	13	13
	PI	4	4	7	7	6	5	2	2	2

## Conclusion

Accurate RUL prediction is at the core of LIBs Prognostics and Health Management (PHM). Particle filter is one of the primary model-driven methods for RUL prediction. However, the particle degeneracy is an inevitable issue, which will lead to a decrease in the prediction accuracy. This study introduced the grey model and PFF to predict the remaining useful life of LIBs. Evaluations were conducted using three performance metrics, RMSE, AE, and PI, to assess the PFF results at different stages (early, middle, and late periods). Probability density distribution ranges were used to express the uncertainty of prediction outcomes. Experimental results on two commonly used datasets indicate that, compared to the traditional PF, the proposed GM-PFF mitigates the risk of particle degeneration, narrows the uncertainty range of prediction results, and exhibits lower errors in predicting the RUL of LIBs. However, the accuracy of particle flow filter predictions is excessively dependent on the initial values of model parameters. Future research will involve introducing different particle flow strategies to predict the RUL of LIBs as well as analyzing and comparing the performance of various PFF types, with the aim of enhancing the accuracy and robustness of PFF predictions.

## Data Availability

The data can be accessed at https://calce.umd.edu/battery-data.
